# The bHLH network underlying plant shade‐avoidance

**DOI:** 10.1111/ppl.13074

**Published:** 2020-02-28

**Authors:** Sara Buti, Scott Hayes, Ronald Pierik

**Affiliations:** ^1^ Plant Ecophysiology, Institute of Environmental Biology Utrecht University Utrecht 3584 CH The Netherlands; ^2^ Centro Nacional de Biotecnología, CSIC Madrid 28049 Spain

## Abstract

Shade is a potential threat to many plant species. When shade‐intolerant plants detect neighbours, they elongate their stems and leaves in an effort to maximise their light capture. This developmental programme, known as ‘shade‐avoidance’ is tightly controlled by specialised photoreceptors and a suite of transcriptional regulators. The basic helix–loop–helix (bHLH) family of transcription factors are particularly important for shade‐induced elongation. In recent years, it has become apparent that many members of this family heterodimerise and that together they form a complex regulatory network. This review summarises recent work into the structure of the bHLH network and how it regulates elongation growth. In addition to this, we highlight how photoreceptors modulate the function of the network via direct interaction with transcription factors. It is hoped that the information integrated in this review will provide a useful theoretical framework for future studies on the molecular basis of shade‐avoidance in plants.

**Abbreviations**
ACE: ACTIVATOR FOR CELL ELONGATION
AIF: ATBS1 (ACTIVATION‐TAGGED BRI1 SUPPRESSOR 1)‐INTERACTING FACTOR
ARF: AUXIN RESPONSE FACTOR
*atbs1‐D*: *activation‐tagged bri1 suppressor 1‐Dominant*
B: blue
BEE: BR ENHANCED EXPRESSION
BEH: BES1‐HOMOLOGUE
BES1: BRI1 EMS‐SUPRESSOR 1
bHLH: basic helix–loop–helix
BIM: BES1‐INTERACTING MYC‐LIKE
BR: brassinosteroid
BRZ: brassinazole
BZR1: BRASSINAZOLE RESISTANT 1
CES: CESTA
CIB: CRYPTOCHROME INTERACTING bHLH
CIL: CIB1‐LIKE PROTEIN
COP1: CONSTITUTIVELY PHOTOMORPHOGENIC 1
cry: cryptochrome
EXP: EXPANSIN
FR: far‐red
GA: gibberellin
HBI1: HOMOLOGUE OF BEE2 INTERACTING WITH IBH1
HFR1: LONG HYPOCOTYL IN FAR‐RED 1
IBH1: ILI1 BINDING BHLH 1
IBL1: IBH1‐LIKE 1
KDR: KIDARI
PAR: PHYTOCHROME RAPIDLY REGULATED
phy: phytochrome
PIF: PHYTOCHROME INTERACTING FACTOR
PIL1: PIF3‐LIKE 1
PRE: PACLOBUTRAZOL RESISTANCE
R: red
TMO7: TARGET OF MONOPTEROS 7
UVR8: UV‐RESISTANCE 8

## Introduction

The basic helix–loop–helix (bHLH) family constitutes one of the largest groups of transcription factors (TFs) in plants with around 160 members in *Arabidopsis thaliana* (Arabidopsis). They are involved in virtually all aspects of plant development from germination onwards, but also in stress tolerance, pathogen defence, nutrient uptake and the transition to reproductive growth. They are required for the formation of trichomes, root hairs, flowers, stomata and the vasculature. In addition to this, many bHLHs have been shown to control stem, root and leaf architecture of plants, with an especially prominent role in developmental plasticity to light cues. Light is one of the most important environmental factors for plants; it is used not only as an energy source but also as an information signal that controls plant development. The quantity, quality, duration and direction of light provides a plant with information on its environment. One of the biggest threats to a plant (especially at the juvenile stage) is over‐topping by neighbours. Upon the detection of shade (or even the threat of shade), many species of plant initiate a developmental programme known as ‘shade‐avoidance’. This developmental programme is orchestrated by direct interactions between photoreceptors and bHLH proteins, cascading into major reprogramming of the transcriptome (Franklin and Whitelam 2005, Martinez‐Garcia et al. 2010).

The bHLHs are defined by a well‐conserved region of approximately 60 amino acids through which they bind DNA. This bHLH domain can be divided into two sections. The N‐terminal ‘basic domain’ is around 15–17 residues long and contains a high proportion of positively charged amino acids, which interact with the negatively charged phosphate backbone of DNA. The HLH domain consists of two amphiphatic α‐helices connected with a variable loop and allows for homo or heterodimerisation with other TFs. Dimerisation is necessary for bHLH binding to the palindromic G‐box sequence motif (CACGTG) or its cognate, the *E*‐box (CANNTG) (Nosaki et al. 2018).

Several studies have aimed to classify the huge bHLH family into smaller subgroups based on their sequence homology (Bailey et al. 2003, Heim et al. 2003, Toledo‐Ortiz et al. 2003, Carretero‐Paulet et al. 2010, Pires and Dolan 2010). The members of these subgroups often have similar or overlapping roles. Many bHLHs heterodimerise with other members of their own or other subgroups and often this is key to their function. With this review, we wish to highlight several of the bHLH interaction networks that have been identified in recent years. We emphasise how these networks shape plant architecture in response to the threat of neighbour shade, particularly in the context of hypocotyl elongation.

The prevalence of bHLH functions has led to their identification in many investigations, and as such many bHLHs have multiple names. To avoid confusion, we have created Table 1 as an overview of the names used for these bHLHs in the literature. We decided to use the bHLH subgroups described in Carretero‐Paulet et al. (2010) as these groupings generally coincide with each family's physiological role.

**Table 1:** List of bHLH transcription factors.

Sub‐family	AT code	bHLH code	Gene name	Abbreviation	Alternative names	Interacts with
14	AT5G08130	bHLH46	BES1‐INTERACTING MYC‐LIKE 1	BIM1		BES1, UVR8, PAR1, PAR2, CRY1, CRY2
14	AT1G69010	bHLH102	BES1‐INTERACTING MYC‐LIKE 2	BIM2		BES1, PAR1
14	AT5G38860	bHLH141	BES1‐INTERACTING MYC‐LIKE 3	BIM3		BES1
16	AT5G39860	bHLH136	PACLOBUTRAZOL RESISTANCE 1	PRE1	BNQ1, TM07L3	ARF6, PAR1, HFR1, AIF2, AIF3, AIF4, IBH1
16	AT5G15160	bHLH134	PACLOBUTRAZOL RESISTANCE 2	PRE2	BNQ2, TM07L2	HFR1
16	AT1G74500	bHLH135	PACLOBUTRAZOL RESISTANCE 3	PRE3	ATBS1, TMO7, BS1	IBH1, AIF1, AIF2, AIF3, AIF4
16	AT3G47710	bHLH161	PACLOBUTRAZOL RESISTANCE 4	PRE4	BNQ3, TM07L1	HFR1, IBH1
16	AT3G28857	bHLH164	PACLOBUTRAZOL RESISTANCE 5	PRE5	TM07L5	IBH1
16	AT1G26945	bHLH163	PACLOBUTRAZOL RESISTANCE 6	PRE6	KIDARI (KDR)	HFR1
18	AT2G43060	bHLH158	ILI1 BINDING BHLH 1	IBH1	ATIBH1	PRE1, PRE3, PRE4, PRE5, BEE2, CIB5, CIL1, CIL2, CIB4, HBI1
19	AT3G05800	bHLH150	ATBS1 (ACTIVATION‐TAGGED BRI1 SUPPRESSOR 1)‐INTERACTING FACTOR 1	AIF1		PRE3, BIN2, BEE2, HBI1
19	AT3G06590	bHLH148	ATBS1 (ACTIVATION‐TAGGED BRI1 SUPPRESSOR 1)‐INTERACTING FACTOR 2	AIF2	RITF1	PRE3, PRE1, CIB2, CIL1, HBI1
19	AT3G17100	bHLH147	ATBS1 (ACTIVATION‐TAGGED BRI1 SUPPRESSOR 1)‐INTERACTING FACTOR 3	AIF3		PRE3, PRE1, CIL1, HBI1
19	AT1G09250	bHLH149	ATBS1 (ACTIVATION‐TAGGED BRI1 SUPPRESSOR 1)‐INTERACTING FACTOR 4	AIF4		PRE3, PRE1, CIL1, HBI1
20	AT4G30410	bHLH159	IBH1‐LIKE 1	IBL1		
20	AT5G57780	bHLH167	PAR1‐RESPONSIVE 1	P1R1		
21	AT2G42870	bHLH165	PHYTOCHROME RAPIDLY REGULATED 1	PAR1	HLH1	BIM1, BIM2, PIF4, PRE1, BEE1, BEE2, BEE3, HBI1, COP1
21	AT3G58850	bHLH166	PHYTOCHROME RAPIDLY REGULATED 2	PAR2	HLH2	BIM1, COP1
21	AT4G30180	bHLH146				
21	AT2G18969	bHLH170				
24	AT2G20180	bHLH15	PHYTOCHROME INTERACTING FACTOR 1	PIF1	PIL5	PHYA, PHYB, HFR1
24	AT1G09530	bHLH8	PHYTOCHROME INTERACTING FACTOR 3	PIF3	PAP3, POC1	PHYA, PHYB, HFR1
24	AT2G43010	bHLH9	PHYTOCHROME INTERACTING FACTOR 4	PIF4	ATPIF4, SRL2	CRY1, CRY2, BZR1, ARF6, PAR1, PHYB, HFR1
24	AT3G59060	bHLH65	PHYTOCHROME INTERACTING FACTOR 5	PIF5	PIL6, A‐PUT2, BHLHB1	CRY1, CRY2, PHYB, HFR1
24	AT5G61270	bHLH72	PHYTOCHROME INTERACTING FACTOR 7	PIF7		PHYB
24	AT4G00050	bHLH16	PHYTOCHROME INTERACTING FACTOR 8	PIF8	UNE10	PHYB
24	AT1G02340	bHLH26	LONG HYPOCOTYL IN FAR‐RED 1	HFR1	RSF1, REP1, FBI1	PRE1, PRE2, PRE4, PRE6, PIF1, PIF3, PIF4, PIF5, COP1, SPA1
24	AT2G46970	bHLH124	PIF3‐LIKE 1	PIL1	PIF2	PHYB
24	AT3G62090	bHLH132	PIF3‐LIKE 2	PIL2	PIF6	PHYB
24	AT4G36930	bHLH24	SPATULA	SPT		
24	AT5G67110	bHLH73	ALCATRAZ	ALC		
24	AT4G28790	bHLH23				
24	AT4G28800	bHLH56				
24	AT4G28811	bHLH119				
24	AT4G28815	bHLH127				
24	AT1G68240	bHLH109				
25	AT1G18400	bHLH44	BR ENHANCED EXPRESSION 1	BEE1		PAR1, CES
25	AT4G36540	bHLH58	BR ENHANCED EXPRESSION 2	BEE2		PAR1, ARF6, AIF1, IBH1
25	AT1G73830	bHLH50	BR ENHANCED EXPRESSION 3	BEE3		PAR1
25	AT2G18300	bHLH64	HOMOLOGUE OF BEE2 INTERACTING WITH IBH 1	HBI1		AIF1, AIF2, AIF3, AIF4, PAR1, ARF6, CRY1, IBH1
25	AT1G25330	bHLH75	CESTA	CES	HAF	BEE1
25	AT4G34530	bHLH63	CRYPTOCHROME‐INTERACTING BHLH 1	CIB1		CRY1, CRY2
25	AT5G48560	bHLH78	CRYPTOCHROME‐INTERACTING BHLH 2	CIB2		CRY1, AIF2
25	AT3G07340	bHLH62	CRYPTOCHROME‐INTERACTING BHLH 3	CIB3	GBOF1	
25	AT1G10120	bHLH74	CRYPTOCHROME‐INTERACTING BHLH 4	CIB4	ACE2	CRY1, IBH1
25	AT1G26260	bHLH76	CRYPTOCHROME‐INTERACTING BHLH 5	CIB5		CRY1, IBH1
25	AT1G68920	bHLH49	CIB1‐LIKE PROTEIN 1	CIL1	ACE1	IBH1, AIF2, AIF3, AIF4
25	AT3G23690	bHLH77	CIB1‐LIKE PROTEIN 2	CIL2	ACE3	IBH1
25	AT1G59640	bHLH31	BIG PETAL	BPE	BPEP, BPEUB, ZCW32	
25	AT2G42300	bHLH48				
25	AT5G50915	bHLH137				
25	AT5G62610	bHLH79				
25	AT3G57800	bHLH60				

## Light signalling

Shade from neighbouring plants strongly reduces photosynthetic capacity. For this reason, many plants respond to shade‐cues through a range of responses including the inhibition of seed germination, the promotion of hypocotyl, petiole and internode elongation, the upward movement of leaves (hyponasty), the inhibition of branching and accelerated flowering (Franklin and Whitelam 2005, Martinez‐Garcia et al. 2010, Casal 2012, Ruberti et al. 2012, Pierik and De Wit 2013).

In direct sunlight, plants are exposed to a small amount of high energy UV‐B light, and roughly equal proportions of blue (B), red (R) and far‐red (FR) light. Plant canopies absorb UV‐B, R and B light whilst they reflect and transmit FR light. Plants are able to perceive these changes of light cues in canopies through an elaborate signalling system composed of several photoreceptors. FR light is reflected from nearby plants before direct shading occurs. Therefore, one of the earliest signals of impending shade is a drop in the ratio of R to FR light. The ratio of R:FR light is detected through the phytochrome photoreceptors. In the model species *A. thaliana*, five phytochrome genes have been identified to encode the respective proteins: phyA, phyB, phyC, phyD and phyE (Casal 2012). Despite their sometimes overlapping roles, phyB plays the dominant role in shade‐avoidance. Phytochromes exist in two forms, an inactive Pr form, which has a peak of absorbance at 660 nm (R light), and the active Pfr form with a maximum absorbance at 730 nm (FR light). The inactive Pr form exists mainly in the cytoplasm, whereas the active Pfr form is largely nuclear. In the nucleus Pfr targets several light‐responsive TFs for degradation. A drop in the ratio of R:FR light promotes an accumulation of the inactive Pr form of phytochrome, which releases these TFs to promote elongation (Casal 2012).

Plants are also able to sense a reduction in B light, once direct shading has occurred. Indeed, low levels of B light can lead to a greater degree of hypocotyl elongation than low R:FR (Pierik et al. 2009). Blue light quantity is sensed through the cryptochromes. In Arabidopsis, the cryptochromes cry1 and cry2 are activated by B light. Like phytochromes, cryptochromes suppress the activity of a number of TFs, many of which overlap between the two classes of photoreceptors (Keller et al. 2011, Pedmale et al. 2016). In the low B light environment of shade, cryptochromes are less active and this releases the suppression of TFs that are then able to drive elongation (Keller et al. 2011, Keuskamp et al. 2011, 2012, Pedmale et al. 2016). Interestingly, there is a synergistic interaction between low B and low R:FR light signals and the presence of both of these factors has more severe effects than either of these alone (de Wit et al. 2016). Recently it was also shown that low R:FR light enhances phototropic bending of the hypocotyl towards blue light (Goyal et al. 2016). It has been suggested that under deep shade, enhanced phototropism helps plants to reach towards gaps in the canopy; maximising light capture (Goyal et al. 2016).

As UV‐B is strongly filtered‐out in plant canopies, the absence of UV‐B may also be considered a shade‐signal (Favory et al. 2009, Hayes et al. 2014). Plants can detect UV‐B radiation through the protein UV‐RESISTANCE 8 (UVR8; Rizzini et al. 2011). Similar to phytochromes and cryptochromes, UVR8 inhibits the action of several elongation‐promoting TFs (Hayes et al. 2014, Liang et al. 2018). In shade conditions, reduced UVR8 activity likely leads to enhanced action of these TFs. In addition to direct inhibition of TFs, UVR8, phytochromes and cryptochromes all suppress the function of an E3‐ligase known as CONSTITUTIVELY PHOTOMORPHOGENIC 1 (COP1). COP1 promotes the activity of several elongation‐promoting TFs (Kim et al. 2014, Sharma et al. 2019) whilst triggering the degradation of those that repress elongation (Podolec and Ulm 2018). COP1 activity is enhanced in shade‐light where it functions as a positive regulator of shade‐avoidance (Pacín et al. 2016).

## bHLH sub‐family groups

### The PIF sub‐family

**Figure 1:**
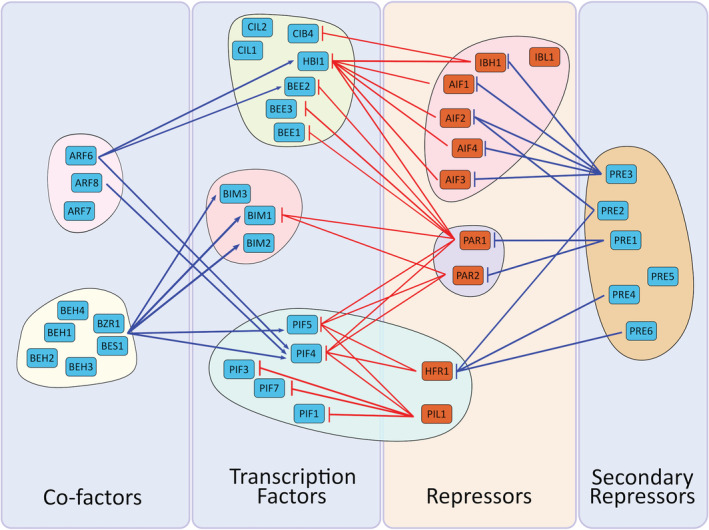
Physical interactions between bHLH sub‐families in the control of hypocotyl elongation under shade. Factors that promote elongation are coloured blue, and those that inhibit elongation are coloured orange. Physical interactions that result in an enhancement of elongation are shown in blue, and those that inhibit elongation are shown in red. The PIF, BIM and BEE sub‐families (excluding HFR1 and PIL1) represent typical bHLH transcription factors that bind to DNA and directly modulate transcription. BIM and PIF activity is enhanced through heterodimerization with BES1/BZR1 family transcription factors. PIFs additionally heterodimerise with ARFs, as do members of the BEE sub‐family. Upon activation, these transcription factors promote the expression of the *AIFs*, *PARs* and *HFR1*/*PIL1*. The protein products of these genes heterodimerise with the transcription factors and inhibit their transcriptional activity. In addition to this primary negative feedback loop, the transcription factors also promote the expression of *PREs*. PREs heterodimerise with the AIFs, PARs and HFR1 and through this they release the transcription factors from repression.

One of the most well‐studied bHLH sub‐families are the PHYTOCHROME INTERACTING FACTORs (PIFs). The PIF sub‐family can be divided based on whether they positively or negatively regulate shade‐avoidance (Fig. 1). Hypocotyl elongation is promoted by PIF1, 3, 4, 5 and 7 and inhibited by PIF3‐LIKE 1 (PIL1) and LONG HYPOCOTYL IN FAR‐RED 1 (HFR1). The founding members of the PIF family, PIF3, was first identified in a yeast‐two‐hybrid (Y2H) screen using the C‐terminus of phyB as bait (Ni et al. 1998). Later research showed that PIF activity is antagonised by several photoreceptors (Monte et al. 2004, Hayes et al. 2014, Ma et al. 2016, Pedmale et al. 2016) and as such they play a central role in light signalling. Shade‐induced hypocotyl elongation is driven by PIF4, PIF5 and PIF7 (Lorrain et al. 2008, Li et al. 2012, de Wit et al. 2016, Hayes et al. 2019) and PIF1 and PIF3 play important roles in day‐length‐dependent hypocotyl elongation (Soy et al. 2012, 2014).

PIF1, 3, 4, 5 and 7 control the expression of a large portion of the genome through directly binding to G‐boxes and *E*‐boxes in the promoters of their target genes (Li et al. 2012, Pfeiffer et al. 2014). In shade‐conditions, these PIFs promote the synthesis of the hormone auxin through upregulating auxin biosynthetic genes (Hornitschek et al. 2012, Li et al. 2012). PIF4 and PIF5 have additionally been shown to promote plant sensitivity to auxin in deep shade (Hersch et al. 2014). This increase in auxin abundance and activity is required for shade‐avoidance.

Similar to PIF1, 3, 4, 5 and 7, PIL1 does have the ability to bind DNA, but it has been argued that its primary mechanism of action is not through direct modulation of gene expression. Instead, PIL1 heterodimerises with the PIFs and thereby blocks PIF action (Li et al. 2014). *PIL1* expression is strongly induced by low R:FR light in a PIF‐dependent manner (Hornitschek et al. 2012) and it, therefore, likely functions in a negative regulatory loop. Accordingly, over‐expression of *PIL1* results in plants with a reduced response to shade (Li et al. 2014, Luo et al. 2014). In contrast to PIL1, HFR1 contains mutations within its bHLH region that render it unable to directly bind DNA. HFR1 does, however, function in a similar manner to PIL1, in that it forms competitive dimers with the PIFs. As *HFR1* expression is also upregulated by PIFs in response to shade, HFR1 constitutes another negative feedback loop to limit PIF function (Sessa et al. 2005, Roig‐Villanova et al. 2006, 2007, Hornitschek et al. 2009). It was recently shown that heterodimerisation between PIF1 and HFR1 promotes the degradation of both proteins (Xu et al. 2017). Additionally, HFR1 and PIL1 can heterodimerise with each other (Luo et al. 2014), but the physiological implications of this interaction remain to be shown.

### The BEE sub‐family

The *BR ENHANCED EXPRESSION* (*BEE*) members were first identified due to their rapid induction by the hormone brassinosteroid (BR; Friedrichsen et al. 2002). Initially, three BEE family members were identified, BEE1‐3 (Friedrichsen et al. 2002), but since then many more have been found; several of which are involved in the light control of plant architecture. HOMOLOGUE OF BEE2 INTERACTING WITH IBH1 (HBI1) was identified based on its homology to BEE2 and its ability to interact with ILI1 BINDING BHLH 1 (IBH1; Bai et al. 2012a; see ‘AIF sub‐family’ below). Another three BEE sub‐family members were designated ACTIVATOR FOR CELL ELONGATION 1–3 (ACE1‐3) also following their identification as IBH1‐interacting factors (Ikeda et al. 2012). ACE1‐3 are also known as CRYPTOCHROME INTERACTING bHLHs (CIBs) and CIB1‐LIKE PROTEINs (CILs), see Table 1 for details. Another member of the BEE sub‐family, CESTA (CES) was discovered through a screen of activation‐tagged T‐DNA insertions (Poppenberger et al. 2011). The over‐expression of *ACE1‐3*, *CIB5* or *CESTA* results in mildly elongated hypocotyls (Poppenberger et al. 2011, Ikeda et al. 2012) suggesting that these bHLHs may require other factors for their full function. In contrast to this, *HBI1* over‐expressors have dramatically elongated hypocotyls (Bai et al. 2012a). There is only limited data available for the phenotypes of BEE sub‐family mutants, and this may be due to redundancy in their function. For example, it has been reported that, *bee1*, *bee2* and *bee3* single and double mutants have no visible phenotype, whereas the triple mutant (*bee1 bee2 bee3*) has short, semi‐dwarf structure (Friedrichsen et al. 2002). The expression of *BEE1‐3* is induced by low R:FR light and the *bee1 bee2 bee3* mutant shows reduced hypocotyl elongation in response to these conditions (Cifuentes‐Esquivel et al. 2013). In addition to controlling cell elongation, several BEE sub‐family members also play an important role in the light‐mediated regulation of flowering transition (Liu et al. 2008, Wang et al. 2019).

BEEs are typical bHLHs that directly regulate transcription. HBI1 has been shown to bind to the G‐box elements of two *EXPANSIN* genes (*EXP1*, *EXP8*), encoding cell wall‐loosening enzymes (Bai et al. 2012a) and CES enhances BR levels through direct activation of BR biosynthesis genes (Poppenberger et al. 2011). Over‐expression of *CES*, *ACE1‐3*, *CIB5* and *HBI1* fused to transcriptional repressor domains results in dwarf plants (Poppenberger et al. 2011, Ikeda et al. 2012, Wang et al. 2018a) consistent with these proteins binding to DNA. The DNA‐binding activity of BEEs is suppressed by several bHLHs (see ‘AIF sub‐family’ and ‘PAR sub‐family’ below).

### The BIM sub‐family

BES1‐INTERACTING MYC‐LIKE 1 (BIM1) was the first member of the BIM sub‐family to be identified. It was isolated through a yeast‐two‐hybrid (Y2H) screen for proteins that interact with BRI1 EMS‐SUPRESSOR 1 (BES1), a key positive regulator of brassinosteroid (BR) signalling (Yin et al. 2005; see ‘BES1/BZR1 family’ below). The BIM sub‐family consists of three closely related proteins, BIM1‐3 (Yin et al. 2005). As with the BEEs, there appears to be a great deal of redundancy between BIMs and it has been reported that only higher‐order knock‐outs show notable phenotypes. Mutants lacking BIM1‐3 (*bim1 bim2 bim3*) have short hypocotyls (Yin et al. 2005) and reduced elongation of hypocotyls and petioles in response to shade light (Cifuentes‐Esquivel et al. 2013). Curiously, over‐expression of *BIM1* had only a minor effect on elongation and a slightly reduced sensitivity to the BR biosynthesis inhibitor brassinazole (BRZ; Yin et al. 2005), indicating that high BIM1 abundance alone is not sufficient to drive elongation. This suggests that BIM1 may require cofactors for its function.

BIM1 binds *E*‐boxes through a highly conserved DNA‐binding domain and heterodimerisation of BIM1 with BES1 enhances this interaction (Yin et al. 2005; See ‘Co‐activators’ below). BIM activity is repressed by light through direct interaction with photoreceptors (Liang et al. 2018, Wang et al. 2018b; see ‘Photoreceptor control of bHLHs’ below) and by heterodimerisation with PAR sub‐family members (Cifuentes‐Esquivel et al. 2013; see ‘PAR sub‐family’ below).

### The PAR sub‐family

The *PHYTOCHROME RAPIDLY REGULATED 1* (*PAR1*) and *PAR2* genes were first characterised based on their rapid upregulation after exposure to low R:FR light (Roig‐Villanova et al. 2006). Over‐expression of either of the genes in this sub‐family leads to a dwarf phenotype and a severely impaired response to shade (Roig‐Villanova et al. 2007). Conversely, plants with a reduced expression of *PAR1* and *PAR2* show a mildly exaggerated response to shade (Roig‐Villanova et al. 2007). As *PAR1* and *PAR2* are strongly induced by low R:FR light this suggests that these factors are negative regulators of shade‐avoidance (Roig‐Villanova et al. 2006, 2007).

PAR1 and PAR2 are small proteins of only 118 residues, and they lack important amino acids in the basic motif responsible for DNA‐binding activity (Roig‐Villanova et al. 2007). How then do the PARs inhibit cell elongation? The secret to PAR action lies in the fact that they heterodimerise with other bHLHs, thereby blocking their DNA‐binding activity. PARs form competitive complexes with members of the PIF, BEE and BIM sub‐family bHLHs, blocking the promotion of cell elongation by these factors (Hao et al. 2012, Cifuentes‐Esquivel et al. 2013, Oh et al. 2014).

### The AIF sub‐family

ATBS1 (ACTIVATION‐TAGGED BRI1 SUPPRESSOR 1)‐INTERACTING FACTOR 1–4 (AIF 1–4) were originally identified in a Y2H screen for PACLOBUTRAZOL RESISTANCE 3 (PRE3) interactors (Wang et al. 2009, see ‘PRE sub‐family’ below). Six members of the AIF sub‐family have been shown to play a role in the control of cell elongation, AIF1‐4, IBH1 and IBH1‐LIKE 1 (IBL1; Zhang et al. 2009, Zhiponova et al. 2014). The *AIFs* are negative regulators of hypocotyl elongation, and *AIF* over‐expressors consequently have a dwarf phenotype (Ikeda et al. 2013).

AIFs are reasonably small bHLHs (150–230 residues), which contain mutations within the conserved DNA‐binding domain (Zhang et al. 2009, Zhiponova et al. 2014). In a similar manner to the PARs, AIF sub‐family members inhibit elongation by forming competitive complexes with typical bHLHs, but in contrast to PARs, AIFs have only been shown to repress BEE sub‐family bHLHs (Zhang et al. 2009, Ikeda et al. 2012, Bai et al. 2012a). The expression of *AIF3*, *AIF4*, *IBH1* and *IBL1* is induced by low R:FR light (Kohnen et al. 2016) and most *AIFs* contain PIF binding sites within their promoters (Oh et al. 2012, Pfeiffer et al. 2014). It is possible therefore that these factors form a negative regulatory loop to repress over‐elongation in shade. It should however be stressed that most of the evidence for the repressive effects of AIFs has been garnered from over‐expressor lines and so future studies using *AIF* knock‐out or knock‐down lines are needed to demonstrate their direct involvement in repressing shade‐avoidance.

### The PRE sub‐family

The PRE sub‐family consists of six members, PRE1‐6. *PRE1* was first isolated in an activation‐tagging screen for plants that could germinate in the presence of the gibberellin (GA) biosynthesis inhibitor, paclobutrazol (Lee et al. 2006). Further characterisation of these lines showed that over‐expression of *PRE1* leads to several traits associated with an enhanced GA response such as elongated hypocotyls and petioles and early flowering (Lee et al. 2006). Over‐expression of the other five members of this group leads to similar phenotypes (Hyun and Lee 2006, Lee et al. 2006).

PREs are in fact involved in numerous biological pathways and as such, they have been isolated in a variety of different screens. This has led to an array of names for PRE family members. *PRE1* was designated *BANQUO* in a study focussing on petal development (Mara et al. 2010). *PRE3* was named after the activation‐tagged line *activation‐tagged bri1 suppressor 1‐Dominant* (*atbs1‐D*) for its ability to overcome the dwarf phenotypes of a mild BR signalling mutant (Wang et al. 2009). *PRE3* is also known as *TARGET OF MONOPTEROS 7* (*TMO7*) and plays a key role in embryonic root initiation (Schlereth et al. 2010). *PRE6* was christened *KIDARI* (*KDR*; ‘tall man’ in Korean) due to the elongated phenotype of *PRE6* over‐expressors (Hyun and Lee 2006).

The expression of *PRE1*, *PRE2*, *PRE4* and *PRE6* is strongly upregulated in low R:FR (Kohnen et al. 2016) and over‐expression of *PREs* enhances elongation in these conditions. For example, *PRE6* and *PRE3* over‐expression leads to longer hypocotyls in low R:FR and in continuous B and FR light, while the knockout line of *PRE6*, *kdr‐1*, has a reduced elongation when compared to the wild‐type (Hyun and Lee 2006, Castelain et al. 2012, Gommers et al. 2017). There are multiple PIF binding sites in the promoters of *PREs* and the increase in *PRE* expression in shade is likely mediated by PIFs (Oh et al. 2012, Bai et al. 2012b, Pfeiffer et al. 2014, Gommers et al. 2017). Downregulation of four *PRE* genes (*PRE1*, *2*, *5* and *6*) in a *pre‐amiR* line resulted in reduced hypocotyl elongation (Oh et al. 2012, Bai et al. 2012b). The expression of *PREs* is also promoted by BR application, likely through the action of BRASSINAZOLE RESISTANT 1 (BZR1; Wang et al. 2009, Zhang et al. 2009; see ‘BES1/BZR1’ below).

The PREs are very small bHLHs (92–94 residues), that have an atypical DNA‐binding domain (Hyun and Lee 2006, Lee et al. 2006, Mara et al. 2010). Instead of directly influencing gene expression, they act as second tier repressors, inhibiting the activity of the AIFs, HFR1 and the PARs, thereby releasing PIFs, BIMs and BEEs to bind DNA (Hyun and Lee 2006, Mara et al. 2010, Hao et al. 2012, Hong et al. 2013, Ikeda et al. 2013). Recently, it was revealed that PRE1‐5 are able to move between cells of the root tip (Lu et al. 2018), but as of yet it is still unclear whether intercellular movement of PREs has any effect on shade‐mediated cell elongation in the shoot.

## Co‐activators

### BES1/BZR1 family

BES1 and BZR1 are two closely related transcription factors that were originally identified in the context of brassinosteroid signalling (He et al. 2005, Yin et al. 2005). They form a subgroup with four other genes, BES1‐HOMOLOGUE 1–4 (BEH1‐4; Yin et al. 2005). BES1 and BZR1 are activated in the presence of BR, where they promote cell elongation and suppress brassinosteroid synthesis in a negative feedback loop (He et al. 2005, Yin et al. 2005). Although BES1/BZR1 family members are not bona fide bHLHs, they do bind to DNA through an unconventional basic helix–loop–helix structure (Nosaki et al. 2018) and act as important co‐factors of the PIFs and BIMs (Yin et al. 2005, Oh et al. 2012, Martínez et al. 2018).

As well as promoting the action of several bHLHs though heterodimerisation, BES1 and BZR1 also bind to the promoters of the majority of bHLHs discussed in this article (Yu et al. 2011, Oh et al. 2012) and it is possible that they contribute to the enhanced expression of many of these genes under shade (Kohnen et al. 2016).

### ARFs

AUXIN RESPONSE FACTORs (ARFs) are a class of TFs that act as the main output of the canonical auxin signalling pathway (Chandler 2016). ARFs are not bHLHs but there are several examples of ARFs binding to bHLHs (Oh et al. 2014) and they are important regulators of bHLH function. The 23 ARFs in Arabidopsis can be split into ‘activating ARFs’ and ‘repressing ARFs’ (Chandler 2016). Of the activating ARFs, ARF6‐8 are particularly required for low R:FR‐induced hypocotyl elongation (Reed et al. 2018). ARF6 and ARF8 act as co‐activators of PIF4, HBI1 and BEE2 to promote cell elongation (Oh et al. 2014).

In addition to co‐activating bHLHs through heterodimerisation, ARFs also bind to the promoters of many shade‐upregulated bHLHs (Oh et al. 2014). For example, the expression of *PRE6* is enhanced by auxin and the *PRE6* promoter is bound by both ARF5 and ARF8 (Zheng et al. 2017). *PRE3* has also been identified as a direct transcriptional target of ARF5 (Schlereth et al. 2010). It may be that the increase in auxin promoted by low R:FR light further activates the bHLH network through stabilization of the ARFs.

### Photoreceptor control of bHLHs

The activity of the bHLH network described above is tightly controlled by photoreceptors. This control allows for rapid changes in gene expression in response to environmental cues. For example, a sudden drop in the ratio of R:FR light triggers an eightfold increase in *PIL1* expression within 15 minutes (rising to over 300‐fold within 45 min; Kohnen et al. 2016). These rapid changes in gene expression are possible due to direct regulation of TF function by photoreceptors.

**Figure 2:**
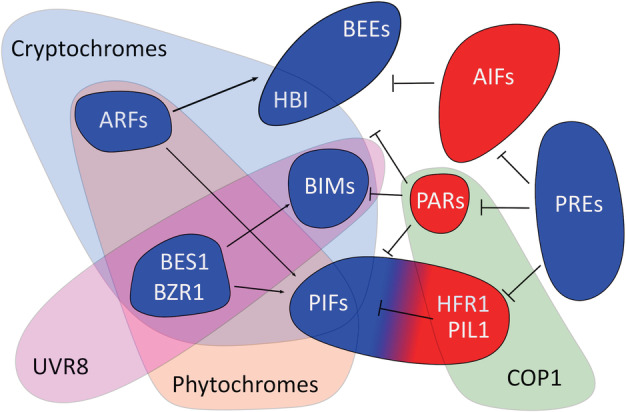
Photoreceptor‐mediated inhibition of the bHLH network. Phytochromes negatively regulate the activity of PIFs and their co‐activators, BES1/BZR1 and the ARFs. BES1/BZR1 are additionally inactivated by UVR8, which also binds to and inhibits the action of BIMs. Cryptochromes antagonise several members of the PIF, BES1/BZR1, ARF, BIM and BEE sub‐families. In conditions where photoreceptor function is reduced, COP1 targets PARs, HFR1 and PIL1 for degradation alleviating their repression of elongation.

The most well‐characterised photoreceptor to bHLH interaction is between phytochromes and the PIFs. When active, phyB inhibits the action of PIF1, 3, 4, 5 and 7 through a number of mechanisms. phyB can directly bind PIFs and inhibit their DNA‐binding activity (Fig. 2; Park et al. 2012). phyB also promotes PIF inactivation through phosphorylation and in some cases by promoting their ubiquitination and degradation (Pham et al. 2018). An inactivation of phyB by low R:FR light results in a rapid release of the PIFs, which then go on to modulate gene expression. Phytochromes do not bind to HFR1 (Fairchild et al. 2000) but they have been shown to bind PIL1, with this interaction leading to an enhanced PIL1 stability (Luo et al. 2014). Recently it was shown that in addition to negatively regulating the PIFs, phyB also binds to members of the BES1/BZR1 family and reduces their DNA‐binding activity (Dong et al. 2019, Wu et al. 2019). Interestingly it was also found that PIF4 can compete with BZR1 for phyB binding (Dong et al. 2019), which could mean that BZR1 activity is enhanced when PIF4 is abundant. Very recently it was demonstrated that photoactive phyB also binds to ARF6 and ARF8 and reduces their ability to bind DNA (Mao et al. 2020). As the interaction of phyB with ARFs and BES1/BZR1 is dependent upon the phyB activation state, it may be that under shade, the activity of these co‐factors of the bHLHs is enhanced, leading to a promotion of elongation.

A recent wave of studies has demonstrated that it is not only phytochromes that control plant architecture through bHLHs but also the cryptochromes. Just over a decade ago it was shown that cry2 binds to the BEE family member CIB1 and that these factors work in conjunction to promote day‐length dependent flowering (Liu et al. 2008). More recently an antagonistic relationship was found between cry1 and PIF4 and PIF5 (Ma et al. 2016, Pedmale et al. 2016). When B light is abundant, cry1 supresses the action of these two bHLHs through directly binding to them. It has been proposed that in low B conditions (such as in deep shade), this repression is lifted and PIFs are able to promote elongation (Pedmale et al. 2016). A similar mechanism has also been put forward for B light‐mediated inhibition of PIF4‐driven growth at warm temperatures (Ma et al. 2016). In addition to the PIFs, cry1 directly binds to HBI1 and BIM1 and blocks their binding to DNA (Wang et al. 2018a, Wang et al. 2018b). cry1 can also interact with several other members of the BEE sub‐family (Wang et al. 2018a) but the physiological relevance of these interactions is presently unclear. In addition to these bHLHs, cry1 also binds to BES1 and BZR1 (Wang et al. 2018b). The interaction between cry1 and BES1 was limited to the active, de‐phosphorylated form of BES1 and resulted in reduced BES1 DNA‐binding (Wang et al. 2018b). ARF6 and ARF8 activity is also supressed by cry1 (Mao et al. 2020) and so in a similar manner to phyB, cry1 also blocks the activation of bHLH co‐factors.

UVR8 also inhibits the action of several bHLHs. Recently, it was shown that UVR8 interacts with BIM1 and reduces its ability to bind DNA (Liang et al. 2018). Furthermore, UVR8 promotes the de‐stabilisation of PIF4 and PIF5 (Hayes et al. 2014) although this is likely an indirect effect through its inhibition of COP1 function (Sharma et al. 2019, Tavridou et al. 2020; see below). UVR8 also directly represses BES1 activity (Liang et al. 2018), which would indirectly reduce PIF and BIM activity. In the absence of UV‐B in true shade, one would therefore expect a reduction of UVR8‐mediated inhibition of these factors.

In shade‐conditions there is an increase in the activity of the E3 ligase COP1 (Pacín et al. 2016), possibly due to reduced suppression of COP1 by phytochromes, cryptochromes and UVR8 (Podolec and Ulm 2018). In low R:FR light, COP1 moves into the nucleus (Pacín et al. 2016). In the nucleus, COP1 de‐stabilises HFR1 and this is likely to reduce the level of negative feedback regulation on the PIFs (Pacín et al. 2016). COP1 also promotes the degradation of several other negative regulators of elongation, including the PAR1, PAR2 and PIL1 bHLH proteins (Duek et al. 2004, Luo et al. 2014, Zhou et al. 2014) and these regulators may therefore be supressed by COP1 in shade. Along with supressing the activity of negative regulators of elongation, COP1 also promotes the activity of PIFs and BZR1 (Kim et al. 2014, Sharma et al. 2019). In dark grown seedlings, PIF3 stability is enhanced by COP1 due to COP1‐mediated suppression of BIN2, a negative regulator of PIF3 (Ling et al. 2017). COP1 may also promote BZR1 activity by a targeted degradation of phosphorylated (inactive) BZR1. It has been argued that degradation of the inactive pool of BZR1 enhances the relative concentration of the active form, and thereby enhances BZR1 function (Kim et al. 2014).

## Conclusions and future perspectives

Tremendous progress has been made in our understanding of the bHLH functions since their discovery in the late 1980s. Several studies have associated this family of TFs with diverse developmental processes but also with biotic and abiotic stress responses. In this review, we focused the attention to specific sub‐families of bHLHs that translate light signals to changes in plant architecture. Different sub‐families were covered and each of them includes several members. Within each sub‐family, several members were found to exhibit partial or even complete redundancy of action. This could be due to gene duplication events that gave rise to multiple copies of the same gene possibly leading to partially diverse function, as supported by the ‘birth‐and‐death’ evolution theory (Nei and Rooney 2005). In order to help the reader to visualise their mode of action we sorted the light response‐associated bHLH subfamilies into three main categories. (1) The typical bHLHs, which have the capability to bind DNA and activate the transcription of genes promoting cell elongation. (2) The atypical or non‐DNA‐binding bHLHs that interfere with the action of the typical bHLHs by forming non‐functional heterodimers. (3) Other non‐DNA‐binding bHLHs, whose function is to form competitive heterodimers with the 2d category of bHLHs, thereby promoting the action of the members of the first category. We have also discussed several common co‐factors of the category 1 bHLHs, as the activity of these co‐factors is intrinsically linked to bHLH function. We summarised these subfamilies in Fig. 1, where we have also added a fourth group of non‐bHLH transcription factors that work closely together with the light‐associated bHLH proteins discussed.

Taken together, we conclude that the response to different light conditions is probably mediated by the equilibrium of several modules of heterodimers formed between bHLH proteins with opposite function in the regulation of elongation. The high redundancy seen in the regulation of this response is striking but might be associated with two important elements that would need to be studied in greater detail. Several genes with homologous functions exert their effects across distinct temporal scales. For example, *PAR1* expression is high in young seedlings, whereas *IBH1* transcripts are much higher in mature tissues (Oh et al. 2014). There is also a spatial component, with tissue‐specific expression of some genes. For example, in the root *AIF3* is enriched in the endodermis and vasculature, whereas *IBL1* is expressed highly in root hairs (ePlant Browser, https://bar.utoronto.ca/eplant/). More spatially distinct analysis of aerial tissues undergoing shade‐avoidance should provide us with a much more detailed understanding of this process.

The direct regulation of many transcription factors by photoreceptors likely contributes to the rapid ability of plants to respond to changes in their light environment. It should, however, be noted that light environments in nature are rarely homogeneous and that they vary across both the long and short term. It may be that the complex feedback regulation on bHLH action acts to buffer plant responses to short term changes.

Further dissection in Arabidopsis of the highly complicated networks of protein interactions and their resulting regulation of transcription, as well as analyses of the output of these interactions in tissue‐, development‐ and time‐specific contexts, could provide valuable knowledge to enhance our understanding of how light responses are controlled. Once these mechanistic details are truly understood, translating such knowledge to crop species may present a sophisticated engineering approach to further increase crop productivity in high plant density cropping systems.

## Author contributions

S.B., S.H. and R.P. developed the concept of the review, S.B. and S.H. wrote the draft manuscript. All authors reviewed, edited and approved the final manuscript.

*Acknowledgements* – Funding was provided by the Dutch Research Council (NWO) to RP (Vici grant nr. 865.17.002) and Marie Skłodowska Curie Action (792624) to SH.

## Data Availability

Data sharing is not applicable to this article as no new data were created or analyzed in this study.
